# 2-(4-Amino­benzene­sulfonamido)-4,6-dimethyl­pyrimidin-1-ium 2-carb­oxy-4,6-dinitro­phenolate

**DOI:** 10.1107/S1600536813005631

**Published:** 2013-03-02

**Authors:** Graham Smith, Urs D. Wermuth

**Affiliations:** a Science and Engineering Faculty, Queensland University of Technology, GPO Box 2434, Brisbane, Queensland 4001, Australia

## Abstract

In the structure of the phenolate salt of the sulfa drug sulfamethazine with 3,5-dinitro­salicylic acid, C_12_H_15_N_4_O_2_S^+^·C_7_H_3_N_2_O_7_^−^, the dihedral angle between the pyrimidine and benzene rings of the cation is 59.70 (17)°. In the crystal, cation–anion hydrogen-bonding inter­actions involving pyrim­idine–carb­oxy N^+^—H⋯O and amine–carb­oxy N—H⋯O pairs give a cyclic *R*_2_^2^(8) motif while secondary N—H⋯O hydrogen bonds between the aniline group and both sulfone and nitro O-atom acceptors give a two-dimensional structure extending in (001).

## Related literature

For background to sulfamethazine and its co-crystals, see: O’Neil (2001 ▶); Caira (2007 ▶); Ghosh *et al.* (2011 ▶). For similar structures, see: Caira (1991 ▶); Lynch *et al.* (2000 ▶); Smith & Wermuth (2013 ▶). For structures of 3,5-dinitro­salicylic acid salts, see: Smith *et al.* (2003 ▶). For graph-set analysis, see: Bernstein *et al.* (1995 ▶).
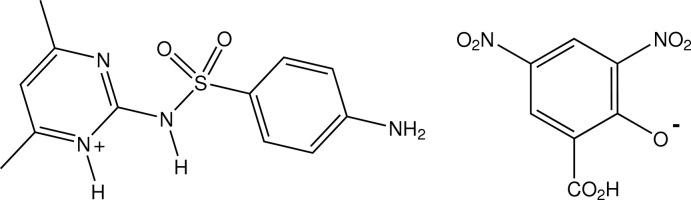


## Experimental

### 

#### Crystal data

C_12_H_15_N_4_O_2_S^+^·C_7_H_3_N_2_O_7_^−^
*M* *_r_* = 506.46
Monoclinic, 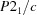

*a* = 8.1691 (3) Å
*b* = 32.0736 (9) Å
*c* = 8.9869 (3) Å
β = 112.258 (5)°
*V* = 2179.23 (15) Å^3^
*Z* = 4
Mo *K*α radiation
μ = 0.22 mm^−1^
*T* = 200 K
0.40 × 0.35 × 0.20 mm

#### Data collection

Oxford Diffraction Gemini-S CCD-detector diffractometer
Absorption correction: multi-scan (*CrysAlis PRO*; Agilent, 2012 ▶) *T*_min_ = 0.918, *T*_max_ = 0.980
14977 measured reflections
4264 independent reflections
3645 reflections with *I* > \2s(*I*)
*R*_int_ = 0.039

#### Refinement

*R*[*F* ^2^ > 2σ(*F* ^2^)] = 0.070
*wR*(*F* ^2^) = 0.158
*S* = 1.10
4264 reflections
318 parameters
H-atom parameters constrained
Δρ_max_ = 0.87 e Å^−3^
Δρ_min_ = −0.51 e Å^−3^

### 

Data collection: *CrysAlis PRO* (Agilent, 2012 ▶); cell refinement: *CrysAlis PRO*; data reduction: *CrysAlis PRO*; program(s) used to solve structure: *SIR92* (Altomare *et al.*, 1993 ▶); program(s) used to refine structure: *SHELXL97* (Sheldrick, 2008 ▶) within *WinGX* (Farrugia, 2012 ▶); molecular graphics: *PLATON* (Spek, 2009 ▶); software used to prepare material for publication: *PLATON*.

## Supplementary Material

Crystal structure: contains datablock(s) global, I. DOI: 10.1107/S1600536813005631/nk2201sup1.cif

Structure factors: contains datablock(s) I. DOI: 10.1107/S1600536813005631/nk2201Isup2.hkl

Supplementary material file. DOI: 10.1107/S1600536813005631/nk2201Isup3.cml

Additional supplementary materials:  crystallographic information; 3D view; checkCIF report

## Figures and Tables

**Table 1:** Hydrogen-bond geometry (Å, °)

*D*—H⋯*A*	*D*—H	H⋯*A*	*D*⋯*A*	*D*—H⋯*A*
N1*A*—H1*A*⋯O11	0.88	1.75	2.617 (4)	168
N2*A*—H2*A*⋯O12	0.78	1.95	2.729 (4)	170
O12—H12⋯O2	0.96	1.52	2.416 (5)	154
N41*A*—H41*A*⋯O51^i^	0.81	2.50	3.248 (5)	153
N41*A*—H42*A*⋯O12*A* ^ii^	0.81	2.46	3.202 (4)	152

Symmetry codes: (i) 
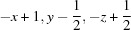
; (ii) 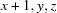.

**Fig. 1.**
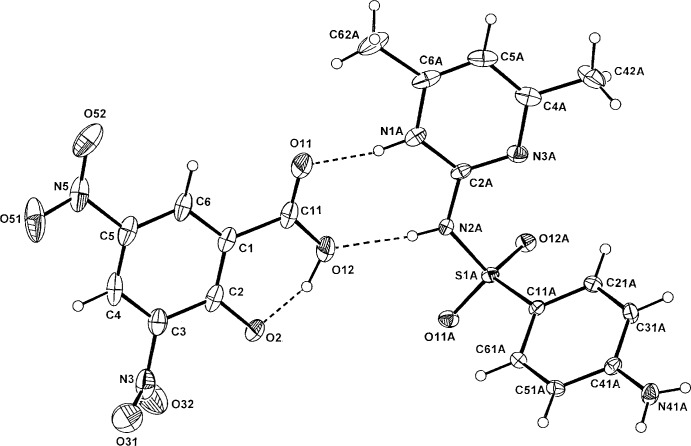
Molecular conformation and atom-numbering scheme for the title compound, with inter-species hydrogen bonds shown as a dashed lines. Non-H atoms are shown as 40% probability displacement ellipsoids.

**Fig. 2.**
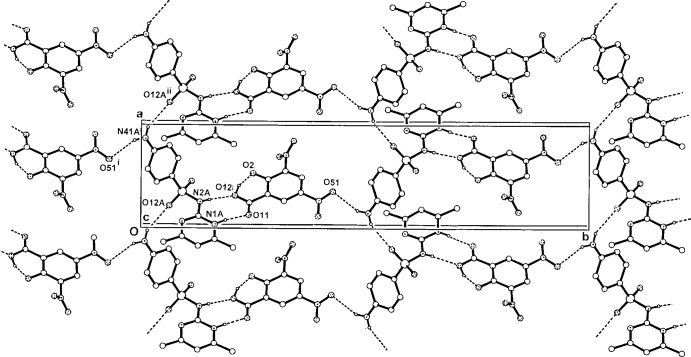
The two-dimensional network structure viewed down c, showing hydrogen-bonding associations as dashed lines. Non-associative H atoms are omitted.

## supplementary crystallographic information

### Comment

The drug sulfamethazine (or sulfadimidine) [4-amino-*N*-(4,6-dimethylpyrimidin-2-yl)benzenesulfonamide] (O'Neil, 2001) has been used as a model for co-crystal formation (Caira, 2007; Ghosh *et al.*, 2011), commonly forming 1:1 adducts with carboxylic acids, predominently the benzoic analogues but including some amides. The structures of a number of these have been reported, e.g. anthranilic acid and 4-aminobenzoic acid (Caira, 1991), 2,4-dinitrobenzoic acid (Lynch *et al.*, 2000), as well as benzamide, 4-hydroxybenzamide and picolinamide (Ghosh *et al.*, 2011). In all of these co-crystals, heterodimers are formed through a cyclic intermolecular hydrogen-bonding motif [graph set *R*^2^_2_(8) (Bernstein *et al.*, 1995)], involving amine N—H···O_carboxyl_ and carboxylic acid O—H···N_pyrimidine_ pairs.

However, there are no examples of the structures of proton-transfer salts of sulfamethazine with carboxylic acids so we looked at the products from the 1:1 stoichiometric reactions with some strong acids. Crystalline materials were obtained from the 5-nitrosalicylic acid and picric acid reactions, namely the anhydrous (1:1) carboxylate and picrate salts, respectively (Smith & Wermuth, 2013). With 3,5-dinitrosalicylic acid (DNSA), the poorly-formed anhydrous 1:1 salt of the title compound, C_12_H_15_N_4_O_2_S^+^ C_7_H_3_N_2_O_7_^-^, was obtained, and the structure is reported herein. DNSA has been particularly useful in providing crystalline proton-transfer salts with both aliphatic and aromatic amines, the majority of which have been picrates, in which an *anti*-related acidic proton is retained on the carboxylic acid group rather than on the phenolic group (Smith *et al.*, 2003).

With the title salt, the phenolate anion is found (Fig. 1), providing a variant of the *R*^2^_2_(8) cation–anion hydrogen-bonding interaction as found in the non-transfer co-crystal structures, the difference arising from the presence of the transferred acid proton on the pyrimidine nitrogen (N1A). The slight asymmetry in the N1A···O and N2A···O hydrogen bond distances [2.622 (5) and 2.732 (4) Å] (Table 1) is comparable with those in the non-transfer co-crystals. In the DNSA anion, the *anti*-related acid proton forms the usual intramolecular hydrogen bond with the phenolate O-atom (Smith *et al.*, 2003). Both H-atoms of the aniline group of the cation participate in intermolecular N—H···O hydrogen-bonding interactions with both sulfone and nitro O-atom acceptors, giving extensions along the *a* and *b* axes respectively, giving a two-dimensional structure lying along (001) (Fig. 2).

In the sulfamethazine cation, the dihedral angle between the pyrimidinium and phenyl rings is 59.70 (17)°, similar to that found in the picrate salt [58.18 (7)°] (Smith & Wermuth, 2013), but significantly smaller than commonly found with the adduct structures, e.g. 70.3 (4)° in the 2,4-dinitrobenzoic acid co-crystal (Lynch *et al.*, 2000). The two interacting pyrimidine–DNSA moieties are close to coplanar [inter-ring dihedral angle 12.2 (2)°].

### Experimental

The title compound was prepared by the reaction of 1 mmol quantities of 4-amino-*N*-(4,6-dimethylpyrimidin-2-yl)benzenesulfonamide (sulfamethazine) with 3,5-dinitrosalicylic in 50 ml of 50% ethanol–water with 10 min refluxing. Partial evaporation of the solvent gave poorly-formed yellow crystal plates (m.p. 457–458 K) from which a specimen was cleaved for the X-ray analysis.

### Refinement

Hydrogen atoms potentially involved in hydrogen-bonding interactions were located by difference methods but their positional and isotropic displacement parameters were subsequently allowed to ride in the refinement with *U*_iso_(H) = 1.2*U*_eq_(N) or 1.5*U*_eq_(O). Other H atoms were included at calculated positions [C—H (aromatic) = 0.93 Å or C—H (methyl) = 0.96 Å] and also treated as riding, with *U*_iso_(H) = 1.2*U*_eq_(C)_aromatic_ or 1.5*U*_eq_ (*C*)_methyl_.

### Crystal data

**Table:**

C_12_H_15_N_4_O_2_S^+^·C_7_H_3_N_2_O_7_^−^	*F*(000) = 1048
*M**_r_* = 506.46	*D*_x_ = 1.544 Mg m^−^^3^
Monoclinic, *P*2_1_/*c*	Melting point = 457–458 K
Hall symbol: -P 2ybc	Mo *K*α radiation, λ = 0.71073 Å
*a* = 8.1691 (3) Å	Cell parameters from 4751 reflections
*b* = 32.0736 (9) Å	θ = 3.1–28.8°
*c* = 8.9869 (3) Å	µ = 0.22 mm^−^^1^
β = 112.258 (5)°	*T* = 200 K
*V* = 2179.23 (15) Å^3^	Plate, yellow
*Z* = 4	0.40 × 0.35 × 0.20 mm

### Data collection

**Table:**

Oxford Diffraction Gemini-S CCD-detector diffractometer	4264 independent reflections
Radiation source: Enhance (Mo) X-ray source	3645 reflections with *I* > \2s(*I*)
Graphite monochromator	*R*_int_ = 0.039
Detector resolution: 16.077 pixels mm^-1^	θ_max_ = 26.0°, θ_min_ = 3.1°
ω scans	*h* = −7→10
Absorption correction: multi-scan (*CrysAlis PRO*; Agilent, 2012)	*k* = −39→39
*T*_min_ = 0.918, *T*_max_ = 0.980	*l* = −11→11
14977 measured reflections	

### Refinement

**Table:**

Refinement on *F*^2^	Primary atom site location: structure-invariant direct methods
Least-squares matrix: full	Secondary atom site location: difference Fourier map
*R*[*F*^2^ > 2σ(*F*^2^)] = 0.070	Hydrogen site location: inferred from neighbouring sites
*wR*(*F*^2^) = 0.158	H-atom parameters constrained
*S* = 1.10	*w* = 1/[σ^2^(*F*_o_^2^) + (0.0472*P*)^2^ + 4.2454*P*] where *P* = (*F*_o_^2^ + 2*F*_c_^2^)/3
4264 reflections	(Δ/σ)_max_ = 0.010
318 parameters	Δρ_max_ = 0.87 e Å^−^^3^
0 restraints	Δρ_min_ = −0.51 e Å^−^^3^

### Special details

**Table:**

Geometry. Bond distances, angles *etc*. have been calculated using the rounded fractional coordinates. All su's are estimated from the variances of the (full) variance-covariance matrix. The cell e.s.d.'s are taken into account in the estimation of distances, angles and torsion angles
Refinement. Refinement of *F*^2^ against ALL reflections. The weighted *R*-factor *wR* and goodness of fit *S* are based on *F*^2^, conventional *R*-factors *R* are based on *F*, with *F* set to zero for negative *F*^2^. The threshold expression of *F*^2^ > σ(*F*^2^) is used only for calculating *R*-factors(gt) *etc*. and is not relevant to the choice of reflections for refinement. *R*-factors based on *F*^2^ are statistically about twice as large as those based on *F*, and *R*- factors based on ALL data will be even larger.

### Fractional atomic coordinates and isotropic or equivalent isotropic displacement parameters (Å^2^)

**Table:**

	*x*	*y*	*z*	*U*_iso_*/*U*_eq_	
S1A	0.33370 (9)	0.09248 (2)	0.49476 (9)	0.0225 (2)	
O11A	0.4293 (3)	0.11377 (7)	0.6420 (3)	0.0308 (7)	
O12A	0.1986 (3)	0.06331 (7)	0.4858 (3)	0.0300 (7)	
N1A	0.0333 (4)	0.16571 (9)	0.1560 (3)	0.0347 (9)	
N2A	0.2414 (3)	0.13221 (8)	0.3706 (3)	0.0279 (8)	
N3A	0.0595 (4)	0.09249 (9)	0.1533 (3)	0.0318 (8)	
N41A	0.8562 (4)	0.00864 (9)	0.2912 (4)	0.0352 (9)	
C2A	0.1081 (4)	0.12918 (10)	0.2241 (4)	0.0271 (9)	
C4A	−0.0789 (5)	0.09197 (13)	0.0121 (4)	0.0394 (11)	
C5A	−0.1668 (5)	0.12839 (15)	−0.0589 (4)	0.0487 (13)	
C6A	−0.1059 (5)	0.16547 (14)	0.0136 (4)	0.0460 (14)	
C11A	0.4832 (4)	0.06863 (9)	0.4269 (4)	0.0209 (8)	
C21A	0.4332 (4)	0.03461 (10)	0.3223 (4)	0.0281 (9)	
C31A	0.5563 (4)	0.01481 (10)	0.2778 (4)	0.0306 (10)	
C41A	0.7336 (4)	0.02831 (9)	0.3364 (4)	0.0257 (9)	
C42A	−0.1332 (6)	0.05060 (14)	−0.0664 (5)	0.0582 (16)	
C51A	0.7806 (4)	0.06280 (10)	0.4394 (4)	0.0279 (9)	
C61A	0.6577 (4)	0.08258 (9)	0.4841 (4)	0.0250 (9)	
C62A	−0.1833 (7)	0.20700 (16)	−0.0541 (6)	0.0709 (17)	
O2	0.5067 (5)	0.25084 (9)	0.7126 (4)	0.0712 (11)	
O11	0.1194 (4)	0.24106 (9)	0.2706 (4)	0.0589 (11)	
O12	0.2967 (4)	0.21077 (8)	0.4967 (4)	0.0606 (10)	
O31	0.8156 (5)	0.34132 (12)	0.8480 (5)	0.0813 (16)	
O32	0.6599 (6)	0.30963 (12)	0.9593 (4)	0.0868 (16)	
O51	0.3439 (7)	0.42888 (10)	0.4234 (4)	0.0993 (19)	
O52	0.1099 (6)	0.39640 (11)	0.2632 (5)	0.0763 (16)	
N3	0.6791 (5)	0.32438 (11)	0.8427 (5)	0.0557 (15)	
N5	0.2539 (7)	0.39791 (10)	0.3752 (5)	0.0568 (16)	
C1	0.3126 (5)	0.28407 (11)	0.4776 (5)	0.0429 (11)	
C2	0.4505 (6)	0.28519 (11)	0.6290 (5)	0.0462 (15)	
C3	0.5295 (6)	0.32354 (12)	0.6872 (5)	0.0456 (14)	
C4	0.4725 (6)	0.36005 (11)	0.6024 (5)	0.0483 (15)	
C5	0.3300 (6)	0.35804 (11)	0.4587 (5)	0.0485 (14)	
C6	0.2494 (6)	0.32095 (11)	0.3902 (5)	0.0464 (15)	
C11	0.2342 (6)	0.24260 (11)	0.4069 (6)	0.0489 (15)	
H1A	0.07050	0.18920	0.20720	0.0420*	
H2A	0.26120	0.15340	0.41760	0.0330*	
H5A	−0.26560	0.12730	−0.15450	0.0580*	
H21A	0.31660	0.02540	0.28290	0.0340*	
H31A	0.52240	−0.00780	0.20800	0.0370*	
H41A	0.83560	−0.01550	0.26240	0.0420*	
H42A	0.95960	0.01430	0.34240	0.0420*	
H43A	−0.17740	0.03360	−0.00190	0.0870*	
H44A	−0.03300	0.03720	−0.07680	0.0870*	
H45A	−0.22420	0.05430	−0.17100	0.0870*	
H51A	0.89660	0.07240	0.47790	0.0330*	
H61A	0.69090	0.10540	0.55300	0.0300*	
H63A	−0.15940	0.22670	0.03180	0.1070*	
H64A	−0.30880	0.20420	−0.11010	0.1070*	
H65A	−0.13110	0.21660	−0.12740	0.1070*	
H4	0.52880	0.38530	0.64130	0.0580*	
H6	0.15680	0.32050	0.29020	0.0560*	
H12	0.38970	0.21900	0.59520	0.0730*	

### Atomic displacement parameters (Å^2^)

**Table:**

	*U*^11^	*U*^22^	*U*^33^	*U*^12^	*U*^13^	*U*^23^
S1A	0.0178 (4)	0.0252 (4)	0.0234 (4)	0.0015 (3)	0.0066 (3)	0.0017 (3)
O11A	0.0251 (11)	0.0396 (13)	0.0253 (12)	0.0024 (10)	0.0069 (10)	−0.0038 (10)
O12A	0.0210 (11)	0.0346 (12)	0.0356 (13)	−0.0023 (9)	0.0122 (10)	0.0024 (10)
N1A	0.0303 (15)	0.0369 (15)	0.0354 (16)	0.0092 (13)	0.0108 (13)	0.0082 (13)
N2A	0.0235 (13)	0.0207 (13)	0.0324 (15)	0.0026 (10)	0.0026 (12)	−0.0014 (11)
N3A	0.0270 (14)	0.0381 (15)	0.0263 (14)	−0.0019 (12)	0.0057 (12)	0.0023 (12)
N41A	0.0307 (15)	0.0290 (14)	0.0512 (18)	0.0007 (12)	0.0215 (14)	−0.0033 (13)
C2A	0.0186 (15)	0.0354 (17)	0.0270 (16)	0.0058 (13)	0.0083 (13)	0.0074 (13)
C4A	0.0289 (18)	0.061 (2)	0.0254 (17)	−0.0095 (17)	0.0071 (14)	0.0030 (17)
C5A	0.031 (2)	0.079 (3)	0.0253 (18)	0.003 (2)	−0.0016 (16)	0.0090 (19)
C6A	0.0322 (19)	0.068 (3)	0.034 (2)	0.0196 (19)	0.0082 (17)	0.0169 (19)
C11A	0.0166 (14)	0.0206 (14)	0.0250 (15)	0.0019 (11)	0.0072 (12)	0.0044 (12)
C21A	0.0212 (15)	0.0263 (16)	0.0347 (18)	−0.0045 (13)	0.0082 (14)	−0.0025 (13)
C31A	0.0294 (17)	0.0221 (15)	0.0396 (19)	−0.0044 (13)	0.0123 (15)	−0.0081 (14)
C41A	0.0272 (16)	0.0236 (15)	0.0284 (16)	0.0042 (13)	0.0129 (14)	0.0064 (13)
C42A	0.060 (3)	0.067 (3)	0.033 (2)	−0.024 (2)	0.001 (2)	−0.004 (2)
C51A	0.0195 (15)	0.0312 (16)	0.0329 (17)	−0.0036 (13)	0.0098 (13)	−0.0001 (14)
C61A	0.0220 (15)	0.0237 (15)	0.0271 (16)	−0.0026 (12)	0.0067 (13)	−0.0022 (12)
C62A	0.065 (3)	0.076 (3)	0.059 (3)	0.040 (3)	0.009 (2)	0.028 (3)
O2	0.074 (2)	0.0400 (16)	0.072 (2)	0.0168 (16)	−0.0036 (18)	0.0058 (15)
O11	0.068 (2)	0.0378 (16)	0.0570 (19)	0.0118 (14)	0.0079 (17)	0.0055 (14)
O12	0.076 (2)	0.0265 (13)	0.0627 (19)	0.0072 (14)	0.0075 (17)	0.0003 (13)
O31	0.055 (2)	0.092 (3)	0.107 (3)	0.006 (2)	0.042 (2)	−0.017 (2)
O32	0.116 (3)	0.083 (3)	0.055 (2)	−0.032 (2)	0.025 (2)	−0.0023 (19)
O51	0.220 (5)	0.0320 (17)	0.057 (2)	−0.005 (2)	0.065 (3)	0.0031 (15)
O52	0.096 (3)	0.067 (2)	0.085 (3)	0.039 (2)	0.056 (2)	0.041 (2)
N3	0.065 (3)	0.0376 (19)	0.074 (3)	0.0023 (18)	0.037 (2)	−0.0111 (18)
N5	0.116 (4)	0.0297 (18)	0.050 (2)	0.010 (2)	0.060 (2)	0.0088 (16)
C1	0.055 (2)	0.0275 (18)	0.053 (2)	0.0116 (17)	0.028 (2)	0.0029 (16)
C2	0.059 (3)	0.0238 (17)	0.065 (3)	0.0117 (17)	0.034 (2)	0.0045 (17)
C3	0.059 (3)	0.036 (2)	0.050 (2)	0.0036 (18)	0.030 (2)	−0.0050 (17)
C4	0.080 (3)	0.0228 (17)	0.064 (3)	−0.0029 (18)	0.052 (3)	−0.0022 (17)
C5	0.085 (3)	0.0319 (19)	0.048 (2)	0.016 (2)	0.047 (2)	0.0092 (17)
C6	0.071 (3)	0.0259 (18)	0.064 (3)	0.0116 (18)	0.050 (2)	0.0054 (17)
C11	0.060 (3)	0.0249 (18)	0.070 (3)	0.0087 (18)	0.034 (2)	0.0028 (18)

### Geometric parameters (Å, º)

**Table:**

S1A—O11A	1.430 (3)	C6A—C62A	1.501 (7)
S1A—O12A	1.426 (3)	C11A—C61A	1.393 (5)
S1A—N2A	1.673 (3)	C11A—C21A	1.397 (4)
S1A—C11A	1.736 (3)	C21A—C31A	1.371 (5)
O2—C2	1.314 (5)	C31A—C41A	1.409 (5)
O11—C11	1.230 (6)	C41A—C51A	1.400 (4)
O12—C11	1.281 (5)	C51A—C61A	1.370 (5)
O31—N3	1.225 (6)	C5A—H5A	0.9300
O32—N3	1.213 (6)	C21A—H21A	0.9300
O51—N5	1.214 (6)	C31A—H31A	0.9300
O52—N5	1.226 (7)	C42A—H45A	0.9600
O12—H12	0.9600	C42A—H43A	0.9600
N1A—C6A	1.352 (5)	C42A—H44A	0.9600
N1A—C2A	1.356 (4)	C51A—H51A	0.9300
N2A—C2A	1.357 (4)	C61A—H61A	0.9300
N3A—C4A	1.342 (5)	C62A—H63A	0.9600
N3A—C2A	1.325 (4)	C62A—H64A	0.9600
N41A—C41A	1.369 (5)	C62A—H65A	0.9600
N1A—H1A	0.8800	C1—C6	1.406 (5)
N2A—H2A	0.7800	C1—C11	1.508 (5)
N41A—H42A	0.8100	C1—C2	1.400 (6)
N41A—H41A	0.8100	C2—C3	1.396 (6)
N3—C3	1.467 (6)	C3—C4	1.378 (5)
N5—C5	1.494 (5)	C4—C5	1.374 (6)
C4A—C5A	1.393 (6)	C5—C6	1.386 (5)
C4A—C42A	1.489 (6)	C4—H4	0.9300
C5A—C6A	1.357 (6)	C6—H6	0.9300
			
O11A—S1A—O12A	120.36 (15)	C6A—C5A—H5A	121.00
O11A—S1A—N2A	101.75 (13)	C4A—C5A—H5A	121.00
O11A—S1A—C11A	108.98 (16)	C11A—C21A—H21A	120.00
O12A—S1A—N2A	108.57 (14)	C31A—C21A—H21A	120.00
O12A—S1A—C11A	108.88 (15)	C41A—C31A—H31A	120.00
N2A—S1A—C11A	107.50 (14)	C21A—C31A—H31A	120.00
C11—O12—H12	110.00	C4A—C42A—H43A	109.00
C2A—N1A—C6A	119.7 (3)	C4A—C42A—H45A	110.00
S1A—N2A—C2A	125.8 (2)	C4A—C42A—H44A	109.00
C2A—N3A—C4A	117.2 (3)	H44A—C42A—H45A	109.00
C6A—N1A—H1A	120.00	H43A—C42A—H44A	110.00
C2A—N1A—H1A	120.00	H43A—C42A—H45A	109.00
C2A—N2A—H2A	121.00	C61A—C51A—H51A	120.00
S1A—N2A—H2A	111.00	C41A—C51A—H51A	120.00
H41A—N41A—H42A	116.00	C51A—C61A—H61A	120.00
C41A—N41A—H42A	117.00	C11A—C61A—H61A	120.00
C41A—N41A—H41A	117.00	H64A—C62A—H65A	109.00
O31—N3—C3	117.6 (4)	C6A—C62A—H64A	109.00
O32—N3—C3	118.8 (4)	C6A—C62A—H65A	110.00
O31—N3—O32	123.6 (5)	H63A—C62A—H64A	110.00
O51—N5—C5	116.2 (4)	H63A—C62A—H65A	109.00
O52—N5—C5	117.8 (4)	C6A—C62A—H63A	109.00
O51—N5—O52	126.1 (4)	C2—C1—C6	120.8 (3)
N2A—C2A—N3A	120.9 (3)	C2—C1—C11	119.3 (3)
N1A—C2A—N3A	123.3 (3)	C6—C1—C11	120.0 (4)
N1A—C2A—N2A	115.8 (3)	O2—C2—C3	120.9 (4)
C5A—C4A—C42A	121.4 (3)	C1—C2—C3	118.3 (3)
N3A—C4A—C42A	116.9 (4)	O2—C2—C1	120.8 (3)
N3A—C4A—C5A	121.7 (4)	N3—C3—C2	118.2 (4)
C4A—C5A—C6A	118.9 (3)	C2—C3—C4	122.0 (4)
N1A—C6A—C5A	118.9 (4)	N3—C3—C4	119.7 (4)
N1A—C6A—C62A	116.9 (4)	C3—C4—C5	117.9 (4)
C5A—C6A—C62A	124.2 (4)	N5—C5—C6	118.3 (4)
C21A—C11A—C61A	119.8 (3)	C4—C5—C6	123.3 (4)
S1A—C11A—C21A	121.0 (3)	N5—C5—C4	118.4 (3)
S1A—C11A—C61A	119.1 (2)	C1—C6—C5	117.5 (4)
C11A—C21A—C31A	119.9 (3)	O11—C11—C1	119.8 (4)
C21A—C31A—C41A	120.8 (3)	O12—C11—C1	115.7 (4)
C31A—C41A—C51A	118.4 (3)	O11—C11—O12	124.5 (4)
N41A—C41A—C51A	120.8 (3)	C3—C4—H4	121.00
N41A—C41A—C31A	120.8 (3)	C5—C4—H4	121.00
C41A—C51A—C61A	120.8 (3)	C1—C6—H6	121.00
C11A—C61A—C51A	120.3 (3)	C5—C6—H6	121.00
			
N2A—S1A—C11A—C61A	89.7 (3)	S1A—C11A—C21A—C31A	−176.2 (3)
O11A—S1A—N2A—C2A	−165.7 (3)	C61A—C11A—C21A—C31A	0.7 (5)
O11A—S1A—C11A—C21A	157.1 (3)	C21A—C11A—C61A—C51A	−0.7 (5)
O12A—S1A—C11A—C21A	24.1 (3)	S1A—C11A—C61A—C51A	176.4 (3)
O12A—S1A—C11A—C61A	−152.9 (3)	C11A—C21A—C31A—C41A	0.0 (5)
O12A—S1A—N2A—C2A	−37.7 (3)	C21A—C31A—C41A—N41A	−179.7 (3)
C11A—S1A—N2A—C2A	79.9 (3)	C21A—C31A—C41A—C51A	−0.9 (5)
O11A—S1A—C11A—C61A	−19.9 (3)	C31A—C41A—C51A—C61A	1.0 (5)
N2A—S1A—C11A—C21A	−93.4 (3)	N41A—C41A—C51A—C61A	179.7 (3)
C2A—N1A—C6A—C62A	179.4 (4)	C41A—C51A—C61A—C11A	−0.2 (5)
C6A—N1A—C2A—N3A	4.6 (6)	C6—C1—C2—O2	178.3 (5)
C6A—N1A—C2A—N2A	−176.9 (3)	C6—C1—C2—C3	−3.2 (7)
C2A—N1A—C6A—C5A	−0.6 (6)	C11—C1—C2—O2	−3.0 (7)
S1A—N2A—C2A—N1A	169.1 (2)	C11—C1—C2—C3	175.5 (4)
S1A—N2A—C2A—N3A	−12.3 (5)	C2—C1—C6—C5	0.8 (7)
C4A—N3A—C2A—N1A	−4.6 (5)	C11—C1—C6—C5	−178.0 (4)
C2A—N3A—C4A—C42A	−179.8 (4)	C2—C1—C11—O11	−176.3 (5)
C4A—N3A—C2A—N2A	176.9 (3)	C2—C1—C11—O12	3.0 (7)
C2A—N3A—C4A—C5A	0.9 (6)	C6—C1—C11—O11	2.5 (7)
O32—N3—C3—C4	125.6 (5)	C6—C1—C11—O12	−178.3 (4)
O31—N3—C3—C4	−52.1 (6)	O2—C2—C3—N3	1.2 (7)
O32—N3—C3—C2	−55.2 (6)	O2—C2—C3—C4	−179.6 (5)
O31—N3—C3—C2	127.1 (5)	C1—C2—C3—N3	−177.4 (4)
O51—N5—C5—C6	−168.2 (5)	C1—C2—C3—C4	1.9 (7)
O52—N5—C5—C4	−167.5 (5)	N3—C3—C4—C5	−178.9 (4)
O52—N5—C5—C6	10.2 (7)	C2—C3—C4—C5	1.9 (7)
O51—N5—C5—C4	14.1 (7)	C3—C4—C5—N5	173.0 (5)
N3A—C4A—C5A—C6A	2.8 (6)	C3—C4—C5—C6	−4.6 (8)
C42A—C4A—C5A—C6A	−176.5 (4)	N5—C5—C6—C1	−174.3 (4)
C4A—C5A—C6A—N1A	−2.9 (6)	C4—C5—C6—C1	3.3 (8)
C4A—C5A—C6A—C62A	177.1 (4)		

### Hydrogen-bond geometry (Å, º)

**Table:**

*D*—H···*A*	*D*—H	H···*A*	*D*···*A*	*D*—H···*A*
N1*A*—H1*A*···O11	0.88	1.75	2.617 (4)	168
N2*A*—H2*A*···O12	0.78	1.95	2.729 (4)	170
O12—H12···O2	0.96	1.52	2.416 (5)	154
N41*A*—H41*A*···O51^i^	0.81	2.50	3.248 (5)	153
N41*A*—H42*A*···O12*A*^ii^	0.81	2.46	3.202 (4)	152
C5*A*—H5*A*···O11*A*^iii^	0.93	2.51	3.408 (5)	163
C51*A*—H51*A*···O12*A*^ii^	0.93	2.46	3.280 (4)	147
C61*A*—H61*A*···O11*A*	0.93	2.56	2.916 (4)	103
C62*A*—H63*A*···O11	0.96	2.51	3.218 (6)	131
C62*A*—H64*A*···O2^iii^	0.96	2.29	2.960 (7)	127

Symmetry codes: (i) −*x*+1, *y*−1/2, −*z*+1/2; (ii) *x*+1, *y*, *z*; (iii) *x*−1, *y*, *z*−1.
